# Blood pressure variability with different measurement methods: Reliability and predictors. A proof of concept cross sectional study in elderly hypertensive hospitalized patients

**DOI:** 10.1097/MD.0000000000016347

**Published:** 2019-07-12

**Authors:** Rosaria Del Giorno, Lorenzo Balestra, Pascal Simon Heiniger, Luca Gabutti

**Affiliations:** a Department of Internal Medicine and Nephrology, Regional Hospital of Bellinzona and Valli, Bellinzona; b Internal Medicine Service, La Carità Hospital, Locarno; c Institute of Biomedicine, University of Southern Switzerland, Lugano, Switzerland.

**Keywords:** ambulatory blood pressure monitoring, beat-to-beat blood pressure monitoring, blood pressure accuracy, blood pressure variability, hospitalized patients, self blood pressure monitoring

## Abstract

Blood pressure variability (BPV) is an independent cardiovascular risk factor in hypertensive patients. The best method for quantifying BPV is still an object of debate. The existence of different BPV patterns, particularly age and arterial stiffness related, is postulated. Our aims were:

i) to compare BPV using different blood pressure (BP) measurement methods
ii) to compare different calculation approaches
iii) to analyze the predictors of BPV.

Cross-sectional study in 108 elderly hypertensive hospitalized patients. Each patient underwent blood pressure measurements with 5 different modalities: 24 hour BP and pulse wave velocity (PWV) monitoring (24hBPM), measurement by nurses or physicians, self-measurement and beat-to-beat monitoring. Differences between maximum and minimum values (ΔBP), averages of the absolute differences between consecutive values (ARV) and coefficients of variation (CV) were calculated.

ΔBP showed the wider values’ dispersion (Δ systolic blood pressure (SBP): 66.4 ± 22.9 and Δ diastolic blood pressure [DBP]: 45.0 ± 13.5 mmHg). ARV and CV were highest with nurses’ measurements (SBP-ARV 9.2 ± 6.2; DBP-ARV 6.9 ± 5.2; SBP-CV 7.6 ± 5.3; DBP-CV 9.6 ± 5.5). The strongest correlation was found comparing physicians’ SBP measurements and 24hBPM ARVs (R2 0.23, *P* <.05). 24hBPM ΔSBP in a multivariate analysis was significantly associated with age (β −3.85, SE 0.83; *P* <.001) and PWV (β 20.29, SE 3.70; *P* <.001). Calcium antagonists were associated with a lower ΔSBP (β −14.6, SE 6.1, *P* <.05) while diuretics and alpha-blockers with a significant increase (β 14.4 SE 5.4, *P* <.01; β 26.9 SE 11.7, *P* <.05).

Age, PWV, diuretics, alpha-blockers, but also measurements obtained by nurses, increase BP variability while calcium antagonists reduce it. BP profiles in elderly in-hospital patients potentially provide important information; they should, however, be interpreted cautiously.

## 1 Introduction

A large and increasing number of studies support the evidence that blood pressure variability (BPV) represents a strong and independent risk factor for cardiovascular diseases (CVD),^[1,2]^ as well as for hypertension-related morbidity and mortality.^[3]^ Furthermore, it has been shown that BPV is associated with the development and severity of target organ damage (involving in particular blood vessels, kidneys, and the heart).^[4,5]^

Fluctuations in blood pressure (BP) can be classified as short-term (assessed as beat-to-beat, minute-to-minute, hour-to-hour or daytime-to-nighttime changes), mid-term (over several days), and long-term (over weeks, months, seasons, or years) variability. Independently from the modality of assessing it, several factors can affect BPV, including in particular gender, age, diabetes, some antihypertensive medications, and obviously the mean blood pressure.^[6,7]^ Autonomic, humoral, neural, vascular, and environmental mechanisms influence in turn BPV.^[8]^ Sustained BPV could also reflect alterations in regulatory mechanisms such as impaired sympathetic drive and baroreflex function (mainly correlated to increased arterial stiffness).^[9,10]^

Knowing that BPV is not only a “background noise” of blood pressure (BP), but an independent risk entity and that evidence is lacking on the best quantitative method for measuring it; more and more questions on BPV reliability arise. Different methods to estimate BPV have in fact been proposed during the last years; among them, the most commonly applied is the average real variability (ARV), which is the average of the absolute differences between consecutive BP readings.^[11]^ ARV has the advantage of taking into account the temporal order of BP measurements, and therefore the BP time series variability. Another parameter frequently used is the coefficient of variation (CV), which is the standard deviation divided by the corresponding mean. However, even if CV provides a good intra-individual estimate of BPV, it does not take into account the order of BP measurements.^[12]^ Lastly, with the intention to quantify the extremes of BP excursions, it was proposed to calculate the difference between the maximum and the minimum BP values (ΔBP), which has the advantage of being independent from the mean.^[13]^

The majority of studies on BPV have been focused on the results of 24-hour ambulatory BP monitoring (ABPM), and comparisons of BPV on other modalities of measurement are lacking. Moreover, BP fluctuations reflect the complex interactions of several, and at least in part, dynamic factors (e.g., environmental, behavioral, drug-related, and dependent on cardiovascular regulatory mechanisms),^[14,15]^ which could produce different patterns of BPV. In this complex panorama, specific BP measurement technique predictor factors for BPV are not available.

This point is potentially relevant, knowing that, in some studies, the association between CVD mortality and BPV was stronger than between the same and mean BP.^[16]^ Hence, the best antihypertensive therapy management should be planned not only considering the mean BP but also taking into account the intra-individual BP fluctuations.^[17]^

In addition, most studies have evaluated factors associated with BPV in young subjects; while in the inpatient real life, elderly are mainly represented; inpatient dynamic in which, at least in the internal medicine wards, the antihypertensive home therapy is often reassessed.

Elderly is a heterogeneous group in which chronic conditions are strongly represented; among those hypertension concerns about 60% of the subjects.^[18]^ Hospitalization *per se* represents an environmental factor, which could largely affect BP, primarily through the stressful experience of adapting to the new setting, which could increase both BP and BP variability.^[19]^ Furthermore, the arterial stiffness increase seen in the elderly, predisposes, in the presence of state of stress, to an excessive pressure response with related large BP fluctuations.^[20]^

Considering the important number of variables potentially affecting BPV in the internal medicine hospital setting, we designed a real-world cross-sectional study aimed at evaluating BPV using different estimation methods (ARV, CV, ΔBP) and different BP measurement strategies: obtained by physicians or nurses; obtained by self-measurement; registered by a 24 hour automated monitor; obtained using a beat-to-beat. The objectives were: i) to compare BPV using different BP measurement methods, ii) to compare different calculation approaches, and iii) to analyze the predictors of BPV, in an elderly in-hospital population.

## 2 Methods

### 2.1 Study design, setting

Single center, cross-sectional study in elderly patients consecutively hospitalized at the internal medicine ward of the teaching Hospital “La Carità” (Locarno, Switzerland) between June 2014 and March 2015. Eligibility criteria for enrollment included:

i) age older than 70 years;
ii) hospitalization in the internal medicine ward not motivated by the hypertension itself.

The exclusions criteria were:

i) inability to understand and to sign the informed consent;
ii) mental illness;
iii) inability to perform BP self-measurements.

The study was carried out according to the Helsinki Declaration and was approved by the Swiss Ethics Committee. All of the participants gave written informed consent.

### 2.2 Study population and procedures

One hundred and eight elderly hospitalized patients were investigated. Each patient underwent 5 modalities of BP measurement:

1) performed by nurses’ staff
2) performed by physicians’ staff
3) performed by the patient himself
4) Performed by a physician using a beat-to-beat
5) Performed over 24 hours by an automated device measuring BP and estimating pulse wave velocity (PWV).

Nurses, physicians, and patients used the same electronic device to measure BP. A Finometer was chosen to register beat-to-beat BP fluctuations. The BP values of the first 2 days of the hospital stay were not considered for the analysis. BP measurements by nurses, physicians and self-measurements by patients were carried out 3 times per day during daytime.

**Figure 1:**
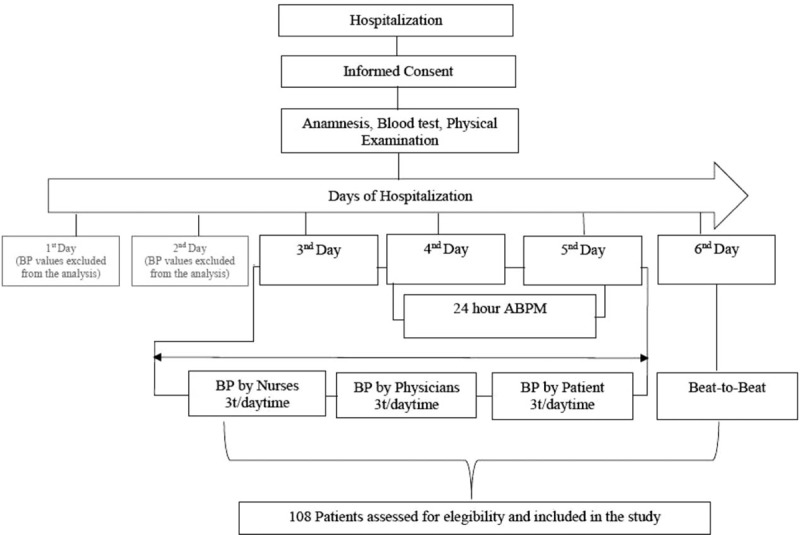
Flow diagram of study and Timeline of the Procedures.

On the 4th day of hospitalization, a 24-hour BP and PWV monitoring using an automated device (Mobil-O-Graph, I.E.M. GmbH, Stolberg, Germany) was performed. See Figure 1 for the flow diagram of the study.

### 2.3 Blood pressure measurements performed by physicians and nurses

BP measurements were performed by physicians and nurses using a, validated and calibrated, automatic oscillometric device (506 N monitor, Criticare System Inc.). The upper non-dominant arm was measured and then, a small cuff was used by circumferences of less than 24 cm, a medium cuff by circumferences between 24 and 34 cm, and a large cuff by circumferences of 35 cm or more. Blood pressure measurements were performed after a comfortable resting of 5 minutes in the seated position. Measurements were performed before taking the medication and without consuming caffeine or tobacco in the preceding 30 minutes. Measurements were performed during daytime at 08h00, 12h00 and 18h00. Recorded systolic blood pressure (SBP) and diastolic blood pressure (DBP) values represented the lowest of 3 different readings measured at 5-minute intervals.

### 2.4 Patient self-measurement

Patients were asked to measure their seated BP thrice, at 08h00, 12h00, and 18h00; after more than 5 minutes of rest each time. BP was measured using the same oscillometric device used by nurses and physicians. Patients were asked to record the results over a 3-day period. The mean of all measurements was calculated for each patient and used for the analysis.

### 2.5 Non-invasive continuous beat-to-beat blood pressure measurement

The Finometer is a device allowing a non-invasive continuous beat-to-beat BP measurement, with a real-time display of the arterial pressure. A calibration of the finger BP on the basis of the brachial BP using an integrated brachial cuff system was performed for every patient. The beat-to-beat blood pressure was registered thanks to small cuffs applied to the fourth finger of the non-dominant hand, which accordingly to a continuous Doppler flow monitoring, inflated and deflated rhythmically extrapolating BP values. After the first 2 minutes dedicated to familiarization, parameters were recorded at 30 seconds, 1, 2, 4, and 6 minutes. At the end of the procedure a “stress induction test” based on standardized questions was performed.

### 2.6 Twenty-four-hour blood pressure monitoring

The 24 hour Blood Pressure Monitoring (24hBPM) was performed using a Mobil-O-Graph (I.E.M. GmbH, Stolberg, Germany); an electronic blood pressure and pulse wave velocity monitor equipped with an arm cuff validated for clinical use. The 24hBPM was applied the fourth day of the study. The cuff was fixed to the non-dominant arm and the device was set to automatically obtain readings every 30 minutes during the day (06:00 AM–10:00 PM) and every hour during the night (10:00 PM–06:00 AM). Eight Mobil-O-Graphs were employed for the study. The supplying company guaranteed the calibration.

### 2.7 Data collection and definitions

Demographic data for each patient were recorded, as well as height, weight, body mass index (BMI), smoking status, patients’ chronic conditions (hypertension, diabetes mellitus, dyslipidemia, chronic kidney disease, ischemic heart disease) and medications in use at home. The weight, in Kg, of the patient was collected with the help of a scale and the height, in cm with a stadiometer. The BMI was calculated with the formula: weight in Kg/(height in m)^2^. The diagnosis of diabetes was considered by the presence of hypoglycemic medications (insulin and/or diabetes oral hypoglycemic agents) or if their fasting blood glucose levels reached 7.0 mmol/L or more. Dyslipidemia was defined by total cholesterol >5.18 mmol/L, triglycerides >1.70 mmol/L, low-density lipoprotein >3.37 mmol/L, high-density lipoprotein <1.04 mmol/L or if the patient was on a lipid-lowering treatment at home. Participants who smoked at least one cigarette per day were classified as current smokers and those who had stopped smoking for more than 3 years were classified as former smokers. Hypertension was defined based on patient's medical history or if the patient was on BP lowering medications over the last 15 consecutive days. Antihypertensive medications in use at home were recorded at admission and categorized into the following groups: renin angiotensin aldosterone system inhibitors (RAAS-I), alpha-adrenergic blocking agents (α-block), calcium channel blockers (Ca-antag), diuretics, beta-adrenergic blocking agents (β-Block), and others. Controlled BP was defined as systolic BP less than 140 mm Hg and diastolic BP less than 90 mm Hg in patients under BP-lowering medications.

### 2.8 Statistical analysis

Variables were shown as median (interquartile range, IQR) or mean ± Standard Deviation (SD); or value and relative frequencies (%) as appropriate. We compared continuous variables and proportions by standard parametric tests and χ^2^ statistics, respectively.

For each measurement method, BPV was estimated for both SBP and DBP, using 3 indices of variability:

I) delta-systolic and diastolic blood pressure (ΔSBP and ΔDBP), expressing for both the difference between maximum and minimum blood pressure values;
II) average real variability (ARV), computed as the average of the absolute differences between consecutive BP measurements;
III) coefficient of variation (CV) obtained by dividing the SD by the average BP level and multiplying by 100.

Correlations between BP obtained with the different measurement methods and the calculated indices of variability were quantified with the Pearson coefficient (expressed as R and relative *P* value).

Subjects were classified in 2 groups of high and respectively low variability, based on the 50^th^ percentile values of each BPV determinant (ΔSBP, ΔDBP, ARV-SBP; ARV-DBP; CV-SBP; CV-DBP) by BP-measurement strategy, and differences among groups were investigated.

Stepwise multiple linear regression analyses were performed in order to explore the covariates associated with BPV. As covariates we considered: gender, age, BMI, dyslipidemia, diabetes, serum creatinine, history of cardiovascular disease, current smoking, pulse wave velocity, height, hypertension, anti-diabetic medication, antihypertensive medication. In the tables, we report the β regression coefficients with their asymptotic standard errors and the associated 2-sided *P* value for the null hypothesis. To explore a possible departure from linearity of the age effect, we repeated the analysis using restricted cubic splines with 3 internal knots.

All statistical analyses were performed using Stata Statistical Software, Release 14, College Station, TX, STATA Corp LP and SPSS 20, SPSS Inc., Chicago, IL. *P* values <.05 (2 tailed) were considered significant.

## 3 Results

A total of 108 patients completed the study and were included in the analysis. Baseline demographic and clinical characteristics of the study population are summarized in Table 1. The median age of the patients was 85.0 years (81.0–89.5), gender was equally distributed (females 52.8%); the BMI was 25.8 kg/m^2^ (22.6–30.3) and 48.2% of the patients had a history of CVD. Patients’ first diagnosis at admission was as follows, n (%): cardiovascular diseases 24 (22.2); pneumonia 20 (18.5); other infectious diseases 15 (13.9); neurological diseases 15 (13.9); gastrointestinal diseases 11 (10.2); oncological diseases 5 (4.6); syncope 4 (3.7); nephrological diseases 3 (2.8); endocrinological diseases 3 (2.8); fractures 3(2.8); falls 2 (1.9); miscellaneous 3 (2.8).

**Table 1:**
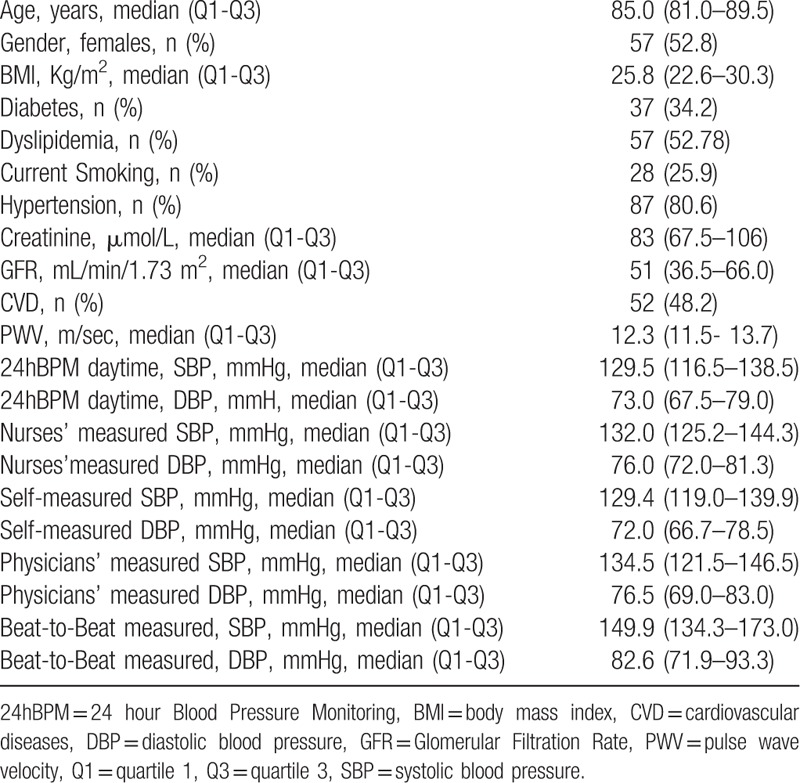
Characteristics of The Study Population.

Mean BP and BPV indices (ΔBP, CV, and ARV) were calculated for each BP measurement method (Table 2). 24hBPM presented the lowest absolute mean BP value and the highest variability for both ΔSBP and ΔDBP (mean ± SD: SBP 128.5 ± 16.8 mmHg; DBP 73.6 ± 9.6 mmHg; ΔSBP and ΔDBP, respectively 66.4 ± 22.9 and 45.0 ± 13.5 mmHg). Analyzing ARV and CV, parameters of variability dependent from the mean, the highest values were obtained with nurses’ BP measurements (SBP-ARV 9.2 ± 6.2 mmHg; DBP-ARV 6.9 ± 5.2 mmHg; SBP-CV 7.6 ± 5.3 mmHg; DBP-CV 9.6 ± 5.5 mmHg), while 24hBPM and Finometer showed lower and similar results (Table 2). The mean BPs of the measurement methods under study were highly correlated for both SDB and DBP (Table 3, panel A). SBP measured by nurses (Pearson coefficient R^2^ 0.72, *P* <.001) and self-assessed (R^2^ 0.62, *P* <.001) showed the highest degree of correlation with 24hBPM. The weakest correlation, on the contrary, was found between SBP obtained by Finometer and 24hBPM (R^2^ 0.52, *P* <.001). Finometer and 24hBPM showed, however, the highest correlation coefficient for DBP (R^2^ 0.53; *P* <.001) (Table 3, panel A). A significant BPV correlation was found comparing ARV of SBP measured by physicians and DBP measured by Finometer, and 24hBPM (R^2^ 0.23 and 0.22, *P* <.05 for both) (Table 3, panel B). Finometer and 24hBPM were significantly associated also for SBP and the determinant of BPV, CV (R^2^ 0.21, *P* <.05). ΔBP was significantly associated for SBP obtained by nurses and 24hBPM (R^2^ 0.21, *P* <.05) and for DBP obtained by patients and 24hBPM (R^2^ 0.21, *P* <.05) (Table 3, panel B). The intra-individual variability of BP was further evaluated based on ΔBP. The subset of patients with higher and lower variability was identified based on the 50th percentile of ΔSBP and ΔDBP. The higher- versus lower-variability groups were investigated across the different BP measurement methods. Descriptive results are shown in Table 4. In Table 4, Panel A, the 2 groups (high- vs. low-variability) of ΔSBP obtained by 24hBPM significantly differed for gender (males 51.7 vs 53.2%; *P* <.05) and pulse wave velocity (13.0 ± 1.3 vs 12.1 ± 1.3 m/s, *P* <.01). Although not statistically significant, age and creatinine were lower in the low-variability group, with respectively 86.3 ± 6.0 vs 84.2 ± 6.2 years, *P* = .077 and 102.2 ± 59.1 versus 96.5 ± 56.6 μmol/L, *P* = .089. Pulse wave velocity values significantly differed also for ΔDBP in 24hBPM (12.8 ± 1.4 vs 12.3 ± 1.4 m/s, *P* <.05) (Table 4, Panel B). The pulse wave velocity was significantly higher also for ΔSBP in the nurses’ BP measurements (12.8 ± 1.3 vs 12.3 ± 1.4 m/s, *P* <.05) (Table 4, Panel A).

**Table 2:**
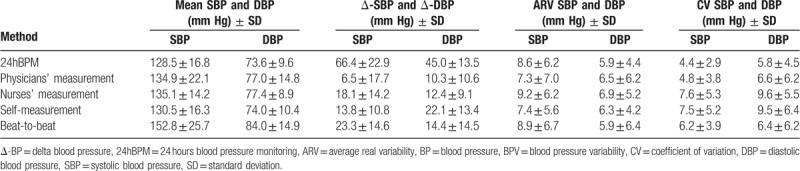
Blood pressure mean (± SD) and variability determinants calculated for each measurement method.

**Table 3:**
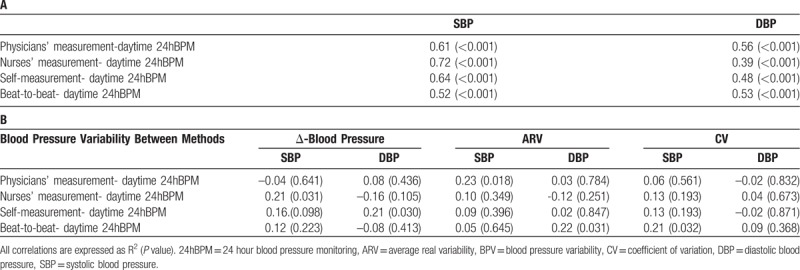
Correlations between blood pressure (Panel A) and variability parameters (Panel B), obtained with the applied measurement methods and daytime 24hBPM.

**Table 4:**
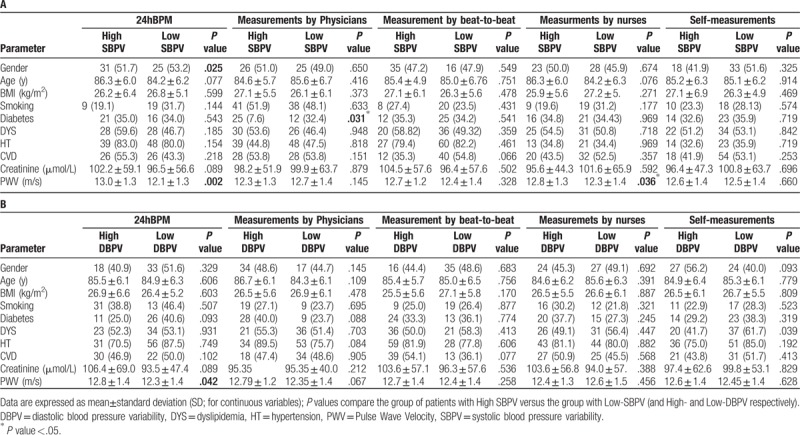
Differences in clinical characteristics of 2 subgroups based on high versus low systolic (Panel A) and diastolic (Panel B) blood pressure variability assessed with different measurements methods.

Multiple linear regression to determine the effect of the potential explanatory variables on different BPV determinants and across different BP measurement methods were also performed (ΔBP, Table 5 , Panel A; ARV Table 5 , Panel B; CV Table 5 , Panel C). 24hBPM ΔSBP was significantly associated with age (β coefficient −3.85, SE 0.83; *P* <.001) and with PWV (β 20.29, SE 3.70; *P* <.001). Similar associations were found for ΔSBP obtained with Finometer, with significant values for age (β -3.58, SE 1.03; *P* = .001) and for PWV (β 17.5, SE 4.64; *P* <.001). In addition, antihypertensive drugs were significantly associated with Finometer ΔSBP, although in opposite directions: calcium antagonists, with a significant reduction (β −14.6, SE 6.1, *P* <.05) while diuretics with a significant increase (β 14.9, SE 5.4, *P* <.01). ΔSBP calculated on the basis of physicians’ measurements was significantly associated with age (β 1.71, SE 0.84, *P* <.05) and with PWV (β -8.25, SE 3.82, *P* <.05). Although not statistically significant, we found a trend towards an association between physicians’ ΔSBP and alpha-blockers (β −36.65, SE 19.31, *P* = .061). Patient ΔSBP was on the contrary significantly associated (β 26.91, SE 11.74, *P* <.05). Considering other determinants of variability we found that ARV-SBP based on nurses’ measurements was significantly associated with the use of beta-blockers (β −3.15, SE 1.47; *P* value .036). ARV-SBP and ARV-DBP based on physicians’ measurements were respectively significantly associated with a positive cardiovascular history (β 3.10, SE 1.48, *P* <.05) and age (β −0.62; SE 1.45; *P* <.05). No other significant associations were found for ARV-SBP and ARV-DBP (Table 5 , Panel B). Considering CV as a determinant of variability, we found a significant association between the use of beta-blockers, patients’ SBP measurements (β–2.68, SE 1.18, *P* <.05); and nurses’ SBP measurements (β 2.90; SE 1.14; *P* <.05). DBP CV based on physicians’ measurement was also significantly associated with age and PWV: β −0.75, SE 0.30, *P* <.05; β 2.70, SE 1.38, *P* = .05. DBP CV based on nurses’ measurements was significantly associated with BMI (β 0.25, SE 0.10, *P* <.05) (Table 5 , Panel C).

**Table 5:**
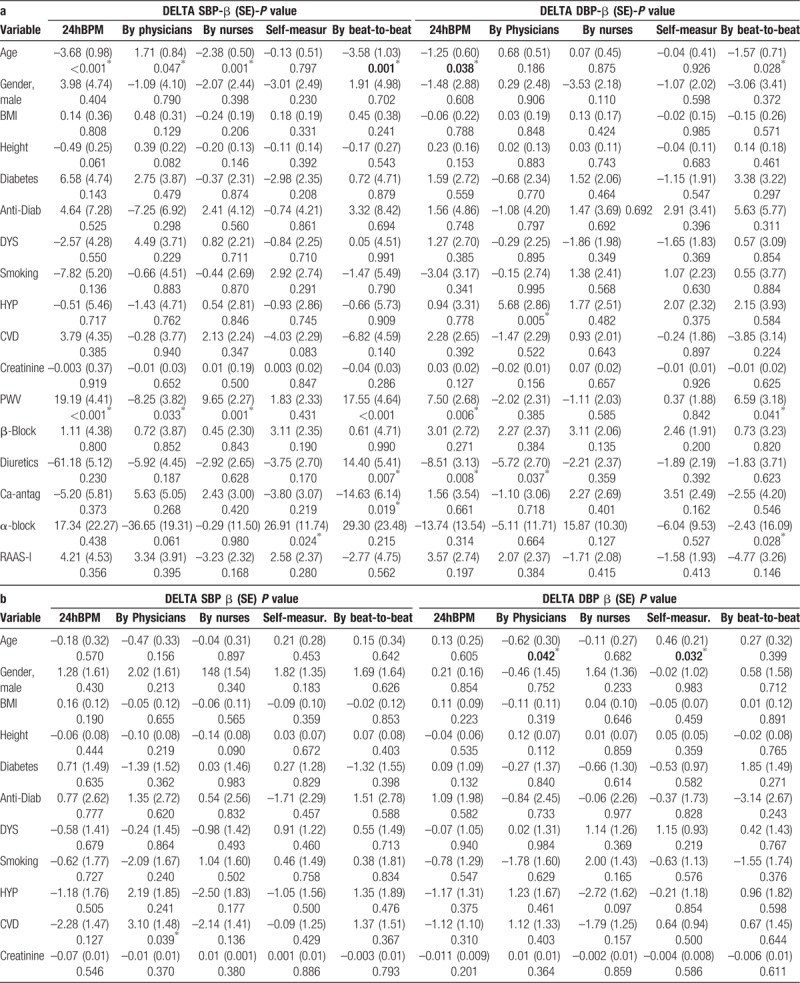
Predictors of systolic and diastolic blood pressure variability according to different blood pressure measurments methods (24hBPM, by physicians, by nurses, self-measurements, by beat-to-beat) and variability determinants (a: Δ-BP, b: ARV, c: CV).

**Table 5 (Continued):**
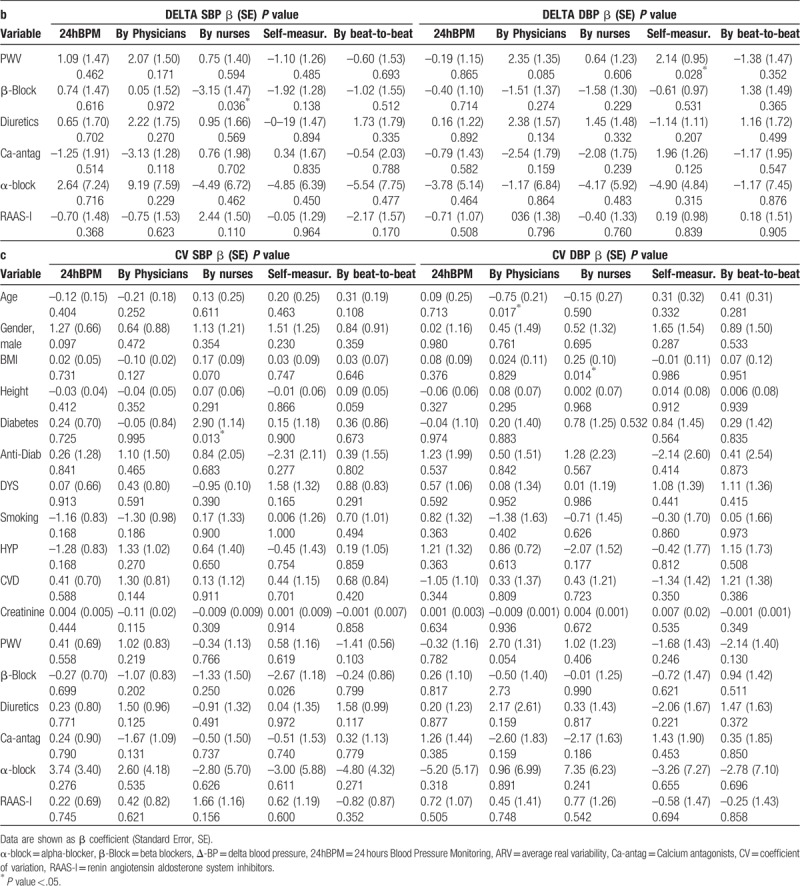
Predictors of systolic and diastolic blood pressure variability according to different blood pressure measurments methods (24hBPM, by physicians, by nurses, self-measurements, by beat-to-beat) and variability determinants (a: Δ-BP, b: ARV, c: CV).

## 4 Discussion

In this study, we confirmed that blood pressure variability is a blood pressure measurement method dependent phenomenon and that the different mathematical determinants of variability used, likely assess peculiar aspects of individual blood pressure behavior.

In our population sample, poor correlation was seen comparing respectively blood pressure obtained with the different methods proposed and the estimators of variability. BP measured by nurses showed the highest variability values compared with measurement performed by physicians and self-measurements.

Although blood pressure variability represents an intriguing research field, with notable clinical and patient outcome implications, 1 important issue still has to be clarified. To date, neither consensus nor gold standard has been published on how and with which method variability should be estimated.

Our proof of concept study is the first one comparing several approaches to calculate blood pressure variability in a specific and distinctive population composed of over 70-year-old in-hospital hypertensive patients. Previous findings did already analyze the topic, highlighting a poor correlation between methods used for variability estimation and investigating the variability agreement between ABPM and beat-to-beat and between ABPM and self-measurements.^[21–24]^ However, our study is the first one in which a full between-methods comparison of blood pressure variability was explored starting from 5 different blood pressure assessment strategies. Furthermore, a wide and exhaustive comparison between blood pressure variability assessment methods was performed. No former study did indeed investigate these variables using 3 different determinants of short-term variability (ARV, CV, and ΔBP). Furthermore, in previous studies, younger populations in heterogeneous environmental circumstances and potentially with several factors influencing punctual BP fluctuations were evaluated.^[23,24]^ Finally, our study is the first one aimed at comparing BPV, also analyzing values obtained with a continuous beat-to-beat noninvasive monitoring system (Finometer).

Our choice to calculate 3 different determinants of short-term variability arises from the attempt to highlight peculiarities of the within-individual differences; peculiarities that can only partially be investigated by BPV determinants dependent on the mean BP.^[25,26]^

In our population, variability calculated on the basis of BP measured by beat-to-beat showed the better systolic and diastolic correlation with values obtained from the 24hBPM. Similarities and differences between BPV determinants were highlighted by the fact that some of them only showed a significant correlation.

Recently, findings of observational studies showed an association between ABPM-BPV and arterial stiffness. This association was more evident focusing on short-term BP variability determinants such as ARV and CV.^[27–28]^ Furthermore, a strong association between arterial stiffness, estimated with a beat-to-beat analyzer in a hypertensive population, and Systolic BPV was shown.^[29]^

Our study confirms, in an in-hospital elderly population, the association between high beat-to-beat BPV and the magnitude of vascular damage measured by pulse wave velocity obtained with an independent method (24hBP and PWV monitor). The fact that arterial stiffness confirms a correlation with the amplitude of BP fluctuations, does not, however, tell us in which way BPV itself impacts on the progression of vascular damage. In our data, moreover, PWV was significantly higher in the High-BPV group. Finally, a rise in PWV correlates with an increase in BP variability for both 24hBPM and measurements obtained by physicians.

Recently, great interest was shown in exploring the contribution of different classes of antihypertensive medications on BPV. In our study, we attempt to provide an estimation of the impact of calcium antagonists, diuretics, alpha-blockers, beta-blockers, and renin angiotensin aldosterone system inhibitors on BPV. As suggested from previous findings, we confirm the association between the use of calcium antagonists and low BPV values. Interestingly, the effect of calcium antagonists on beat-to-beat BP variability was not previously highlighted. Furthermore, increasing the interest in the topic, it was shown in a previous study, that BPV reduction related to calcium antagonist treatment was associated with a concomitant improvement in baroreflex sensitivity, suggesting this mechanism as one of the components of BP stabilization.^[30]^ Diuretics and, as recently shown, alpha- blockers, confirm on the contrary an association with higher BPV values.^[31]^

Moreover increasing evidence suggests a relationship between BPV and, falls, symptomatic hypotension and syncope, mostly in elderly people.^[32,33]^ However, in our study, due to the limited number of patients admitted for the cited diagnoses, we were unable to conduct a targeted sub-analysis. For the same reason, even if some diagnoses justifying hospital admission, especially infections, could impact BPV, we did not investigate their potential role.

We have to acknowledge some limitations of our study. Firstly, the observational design, which does not allow to conclude definitively about causality. Moreover, some strong associations found in our data could not be recognizable in younger patient or in population samples with other characteristics. As a consequence of structural vessel changes, diminished baroreflex sensitivity and enhanced response to sympathetic activation related to increased arterial stiffness, elderly patients represent, in fact, a population with a non-linear and extremely variable pattern of arterial aging and BP behavior. ^[34]^ Moreover, our study was conducted in hospitalized patients; in a setting far from the patient's everyday life in which blood pressure shows a peculiar pattern, related to several influencing factors (e.g., stress generating circumstances, prescription of drugs influencing BP,…), susceptible to impact on reliability and variability and associated with a higher prevalence of symptomatic and asymptomatic orthostatic hypotension.^[35]^

Finally, even if we did not consider the BP values of the first 2 days, to reduce in particular the impact on BP of the adrenergic response to environmental adaptation and the magnitude of white coat hypertension, we cannot exclude a residual hospital mediated “stress-response”.

In this fascinating but still cloudy research field, we can conclude on 1 hand that, individual characteristics and antihypertensive treatments are susceptible to producing peculiar blood pressure variability patterns and on the other that, the way blood pressure is measured and variability is calculated play a significant role. Age, PWV, diuretics, alpha-blockers, but also measurements obtained by nurses, increase blood pressure variability while calcium antagonists reduce it. The cited factors of variability are highly prevalent in the in-hospital internal medicine population; setting in which antihypertensive treatments are often readapted. Blood pressure profiles obtained on the wards, potentially provide important information; they are however difficult to interpret and influenced by numerous biases. A unique consensus about the way of measuring blood pressure variability and their significance as a function of the patients’ characteristics would be helpful.

## Acknowledgment

We would like to thank Anna Zasa MSc for data managing.

## Author contributions

**Conceptualization:** Rosaria Del Giorno, Lorenzo Balestra, Pascal Simon Heiniger, Luca Gabutti.

**Data curation:** Rosaria Del Giorno, Lorenzo Balestra, Pascal Simon Heiniger, Luca Gabutti.

**Formal analysis:** Rosaria Del Giorno, Luca Gabutti, Lorenzo Balestra.

**Investigation:** Pascal Simon Heiniger, Luca Gabutti, Lorenzo Balestra.

**Methodology:** Rosaria Del Giorno, Luca Gabutti.

**Writing – original draft:** Rosaria Del Giorno, Lorenzo Balestra, Pascal Simon Heiniger.

**Writing – review & editing:** Rosaria Del Giorno, Lorenzo Balestra, Luca Gabutti.
